# The Diagnostic Role of Uric Acid to Creatinine Ratio for the Identification of Patients with Adverse Pulmonary Embolism Outcomes

**DOI:** 10.3390/diagnostics12010193

**Published:** 2022-01-14

**Authors:** Konstantinos Bartziokas, Christos Kyriakopoulos, Dimitrios Potonos, Konstantinos Exarchos, Athena Gogali, Konstantinos Kostikas

**Affiliations:** Respiratory Medicine Department, Faculty of Medicine, University of Ioannina, 45500 Ioannina, Greece; ckyriako123@gmail.com (C.K.); jimpotonos@yahoo.gr (D.P.); kexarcho@gmail.com (K.E.); athenagogali@yahoo.com (A.G.); ktkostikas@gmail.com (K.K.)

**Keywords:** uric acid to creatinine ratio, pulmonary embolism, diagnosis, hospitalization, mortality, prognosis

## Abstract

Background: Uric acid (UA) is the final product of purine metabolism and a marker of oxidative stress that may be involved in the pathophysiology of cardiovascular and thromboembolic disease. The aim of the current study is to investigate the potential value of UA to creatinine ratio (UA/Cr) as a diagnostic tool for the outcome of patients admitted with acute pulmonary embolism (PE) and the correlations with other parameters. Methods: We evaluated 116 patients who were admitted for PE in a respiratory medicine department. PE was confirmed with computed tomography pulmonary angiography. Outcomes evaluated were hospitalization duration, mortality or thrombolysis and a composite endpoint (defined as mortality or thrombolysis). Patients were assessed for PE severity with the PE Severity Index (PESI) and the European Society of Cardiology (ESC) 2019 risk stratification. Results: The median (interquartile range) UA/Cr level was 7.59 (6.3–9.3). UA/Cr was significantly associated with PESI (*p <* 0.001), simplified PESI (*p =* 0.019), and ESC 2019 risk stratification (*p <* 0.001). The area under the curve (AUC) for prediction of 30-day mortality by UA/Cr was 0.793 (95% CI: 0.667–0.918). UA/Cr levels ≥7.64 showed 87% specificity and 94% negative predictive value for mortality. In multivariable analysis UA/Cr was an independent predictor of mortality (HR (95% CI): 1.620 (1.245–2.108), *p <* 0.001) and composite outcome (HR (95% CI): 1.521 (1.211–1.908), *p <* 0.001). Patients with elevated UA/Cr levels (≥7.64) had longer hospitalization (median (IQR) 7 (5–11) vs. 6 (5–8) days, *p =* 0.006)), higher mortality (27.3% vs. 3.2%, *p =* 0.001) and worse composite endpoint (32.7% vs. 3.4%, *p <* 0.001). Conclusion: Serum UA/Cr ratio levels at the time of PE diagnosis are associated with disease severity and risk stratification, and may be a useful biomarker for the identification of patients at risk of adverse outcomes.

## 1. Introduction

Pulmonary embolism (PE) covers a wide variety of clinical conditions, which range from asymptomatic, coincidentally revealed subsegmental thrombus found on chest CT scan to pressure-dependent PE complicated by multisystem organ failure and cardiogenic shock [1]. Pulmonary thromboembolism is a serious cardiovascular disease, causing a considerable level of morbidity and mortality. Hemodynamic status, concomitant comorbidities, and dysfunction of the right ventricle are predictors of short-term mortality. Indeed, 30-day mortality rates in patients stratified by the European Society of Cardiology (ESC) classification into high, intermediate-high, and intermediate-low risk groups were found to be 22%, 7.7%, and 6.0%, respectively [2]. Several clinical decision rules have been developed for the evaluation of the pretest clinical probability of PE [3], the most popular being the Well’s rule [4] and the Geneva score [5], which has been revised in 2006 [6].

Oxidative stress upholds a crucial role in the pathophysiologic mechanism of various diseases [7]. Levels of uric acid (UA), the final oxidation product of purine metabolism, arise in conditions of impaired oxidative metabolism, namely cardiovascular disease, idiopathic pulmonary arterial hypertension, chronic thromboembolic pulmonary hypertension, metabolic syndrome, diabetes mellitus and obesity sleep apnea syndrome (OSAS) [8,9,10,11,12,13]. Hyperuricemia has been associated with increased mortality and is considered as an independent predictor of death in patients at high risk of cardiovascular disease [14]. High serum UA levels have long been known to indicate poor prognosis in certain acute diseases [15].

Previous studies have evaluated the role of serum uric acid to creatinine ratio (UA/Cr) and demonstrated the association with the presence of COPD, lung function, COPD severity, dyspnea, disease progression and prediction of exacerbation risk [16,17,18]. UA/Cr has also been associated with hypertension, metabolic syndrome, type 2 diabetes and malignancy [17,19,20].

The aim of the present study was to investigate the potential diagnostic value of serum UA/Cr in the identification of patients with worse prognosis, including adverse outcomes in patients admitted to hospital due an acute PE.

## 2. Materials and Methods

### 2.1. Study Design

Subjects objectively diagnosed with acute pulmonary thromboembolism, according to current guidelines, and those age >18 years, were included in this observational study between January 2019 and December 2020. The study was conducted at the Respiratory Medicine Department of University Hospital of Ioannina that serves as a referral center for patients with suspected pulmonary thromboembolism. This study was conducted in accordance with the amended Declaration of Helsinki and was approved by the Institutional Review Board (Approval number: 27947, approval date 15 October 2019). Informed consent was obtained from all individuals involved in the study. The study outcomes did not affect the future management of the patients, and the authors declare that the patient’s personal data have been secured.

### 2.2. Subjects and Setting

The study inclusion criteria included symptomatic pulmonary thromboembolism confirmed by computed tomography pulmonary angiography, demographic data, troponin, systolic pressure and pulse on admission. Patients were excluded from the study if they met any of the following criteria: (1) referral to our hospital after the beginning of treatment; (2) end-stage renal failure, presence of sepsis, acute coronary syndromes, acute cerebrovascular disease, acute or chronic aortic dissection, acute or chronic infectious diseases, acute or inflammatory diseases such as acute myocarditis and/or pericarditis; (3) any conditions affecting lactic acid (LA) and blood gas analysis, such as severe anemia or metabolic acidosis; and (4) any medication use, at the time or during the month before the baseline examination, that might alter UA levels and/or LA metabolism, such as metformin, acetaminophen, and amoxicillin or consumption of great amount of alcohol.

Risk groups based on early mortality rate were stratified according to the 2019 ESC guidelines [1] and PESI and simplified PESI scores [21,22]. Patients were classified as being at high, intermediate–high, intermediate–low, or low risk, according to the ESC classification. Patients were classified by a numeric number and/or class from I to V (PESI alone) according to the PESI and simplified PESI. Risk adjusted therapy was administered based on these risk classifications. For the evaluation of the pretest clinical probability of PE, Wells rule [4] and the Geneva score [5] were calculated during the initial visit to the emergency department.

Thirty-day all-cause mortality was accepted as the primary end point based on in-hospital deaths or civil registries, as well as duration of hospital stay. Adverse composite outcomes included mortality and clinical conditions that would result in death unless treated with following strategies recommended by the ESC guidelines [1]: (1) thrombolysis, (2) thrombectomy, or (3) extracorporeal membrane oxygenation (ECMO).

Treatment decisions were made by the physicians caring for the subject according to the current ESC guidelines and not influenced by the study protocol. All patients completed follow-up at one month after enrolment. Follow-up included one telephone interview and one surveillance face-to-face evaluation during the one month of study participation in the out-patient clinic.

### 2.3. Biochemical Analysis

Fasting venous blood samples were taken from an antecubital vein with minimal stasis on admission and were centrifuged within 15 min of collection. Troponin-T or hs Troponin-T levels were determined by a quantitative electrochemiluminescence assay (cut-off value < 0.010 ng/mL and cut-off value 0.014 ng/mL, respectively). Serum TnI concentrations were performed on the ADVIA Centaur XP immunoassay analyzer (Siemens Healthcare Diagnostics Inc., Tarrytown, NY, USA). Plasma D-dimer levels were assessed using the BCS XP coagulation analyzer (Siemens Healthcare Diagnostics, Marburg, Germany). Serum UA was measured by enzymatic method (Olympus AU640; Hamburg, Germany) using an enzymatic spectrophotometric method, during the first diagnosis of pulmonary embolism. The normal range of serum uric acid levels was 2.4–6.0 mg/dL (females) and 4.4–7 mg/dL (males), and values are expressed as mg/dL (milligrams per deciliter). The normal range of serum creatinine was between 0.6 and 1.2 mg/dL. Blood cell enumeration and white blood cell differential counts were performed in an automated hematology analyzer (Sysmex K-4500, Roche Diagnostics, Kobe, Japan). All samples were analyzed within 2 h following blood collection without storage. Arterial LA levels were assessed by a sample of arterial blood gases (ABGs), during the initial visit to the emergency department. Values are expressed as mmol/L (millimoles per liter) for LA. ABGs samples were collected from the radial artery in heparinized syringes and gauged with auto-analyzer (ABL3000 auto-analyser Radiometer Co., Tokyo, Japan).

### 2.4. Statistical Analysis

The Kolmogorov–Smirnov and Shapiro–Wilk tests were used to assess conformity with a normal distribution. Categorical values were analyzed using the χ2 test or Fisher’s exact test (chi-square test) as appropriate and were reported as *n* (%). Continuously measured variables were compared by Student’s *t*-test when normally distributed or by the Mann–Whitney U (2 categories) and Kruskal-Wallis (>3 categories) test for non-normally distributed variables. Continuous parameters with normal distribution are reported as means ± SD, while those without normal distribution are reported as median (interquartile range). Correlations between the individual parameters were calculated using the Pearson or Spearman rank correlation coefficients (r_s_) as appropriate. Multivariable logistic regression analysis was performed to assess the relationship between independent variables and 30-day mortality, hospitalization days and adverse outcome. Prognostic factors with a *p* value significance of <0.05 in the univariate analysis were entered in the multivariate model as a stepwise descending method. The level of statistical significance was set at *p*-value < 0.05. All statistical analyses were performed using SPSS for Windows 26.0 (SPSS, Chicago, IL, USA) software and MedCalc version 12.

## 3. Results

**Figure 1:**
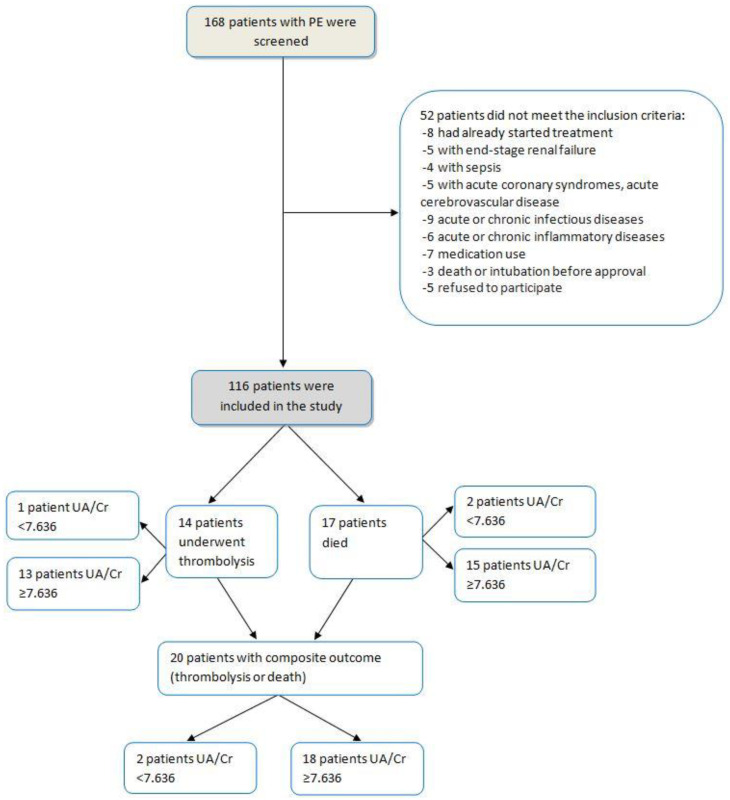
Flow chart of the study participants.

We evaluated 168 consecutive patients admitted for PE and 116 (69.1%) were eligible for inclusion in the present study. The flow chart of study participants is shown in Figure 1. The mean age ± SD of the patients was 53.47 ± 7.87 years and 38.8% were males. The demographic characteristics of the 116 patients who were included in data analysis are presented in Table 1. Patients were divided into two groups according to the presence of serum uric acid to creatinine levels above or below the best value for mortality received through the receiver operating characteristic analysis (≥7.636, *n* = 55 or < 7.636, *n =* 61, respectively). Age and gender distribution were the same in the two groups (*p =* 0.132 and 1.000, respectively). Patients with higher serum UA/Cr had higher respiratory rate (*p <* 0.001), higher PCO_2_ (*p <* 0.001), higher lactic acid (*p =* 0.002) and higher d-dimers (*p =* 0.013). Moreover, cardiopulmonary disease, defined as heart failure or chronic pulmonary disease, was more frequent in patients with higher UA/Cr levels; 8 (13%) vs. 22 (40%), *p =* 0.004.

### 3.1. Association of UA/Cr with PESI Score, Simplified PESI and ESC Risk-Stratification

**Figure 2:**
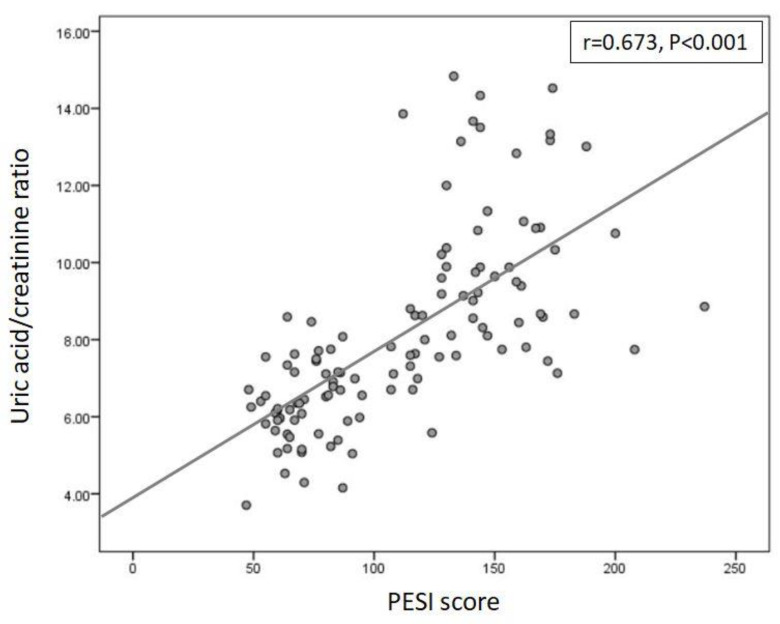
Correlation of uric acid to creatinine ratio levels with PESI score.

**Figure 3:**
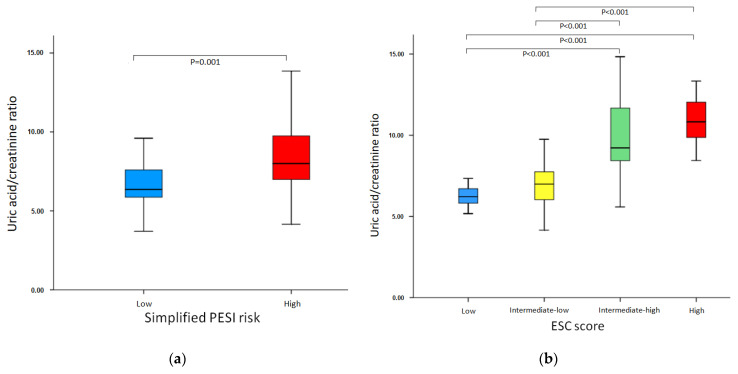
(**a**) Association of Uric acid to creatinine ratio levels with simplified PESI risk-stratification. (**b**) Association of Uric acid to creatinine ratio levels with simplified ESC 2019 risk-stratification.

Serum median (IQR) uric acid to creatinine ratio (UA/Cr) level was 7.59 (6.34–9.35). UA/Cr levels were correlated with PESI score (*r* = 0.673, *p <* 0.001, Figure 2). Serum UA/Cr levels were 6.35 (5.81–7.63) in low (*n* = 31, 26.8%); and 8.00 (6.94–9.81) in high-risk group (*n* = 85, 73.2%) based on simplified PESI risk-stratification (*p* < 0.001) (Table 1, Figure 3a).

Serum UA/Cr levels were 6.21 (5.77–6.77) in low (*n =* 18, 15.5%); 6.98 (5.98–7.75) in intermediate-low (*n =* 55, 47.4%); 9.22 (8.31–12.00) in intermediate-high, (*n =* 35, 30.2%); and 10.82 (9.62–12.60) in high-risk group (*n =* 8, 6.9%) based on 2019 ESC risk-stratification (*p <* 0.001) (Table 1, Figure 3b).

### 3.2. Association of UA/Cr Levels with Hospitalization Outcome

UA/Cr levels were correlated with duration of hospitalization (*r* = 0.382, *p <* 0.001). Patients with elevated UA/Cr levels (≥7.636) had longer hospitalization (median (IQR) 7 (5–11) vs. 6 (5–8) days, *p =* 0.006)), higher mortality (%) (27.3 vs. 3.2, *p =* 0.001) and worse composite endpoint (%) (32.7 vs. 3.4, *p <* 0.001) (Table 2).

### 3.3. Associations of Demographic and Clinical Variables with Duration of Hospitalization, 30-Day Mortality and Adverse Composite Outcome

Multivariate linear regression analysis, as a stepwise descending method with the variables that were significant factors in the univariate analysis, revealed that duration of hospitalization was associated with elevated WBCs (*p* < 0.001), elevated platelets (*p* = 0.002) and higher UA/Cr (*p* = 0.003) (Table 3).

In univariate analyses, 30-day mortality correlated with malignancy(*p <* 0.001), thrombophilia (*p =* 0.036) RV dysfunction (*p =* 0.012), higher respiratory rate (*p =* 0.005), higher PCO_2_ (*p <* 0.001),higher lactic acid (*p =* 0.002), elevated WBCs (*p =* 0.002), hyponatremia (*p <* 0.001), higher CRP (*p <* 0.001), higher PESI (*p =* 0.001) and ESC score (*p <* 0.001), high sPESI risk (*p =* 0.036) and higher UA/Cr (*p <* 0.001). However, age, sex, platelets, low blood pressure, cardiopulmonary disease and heart rate did not affect 30-day mortality.

Multivariate logistic regression analysis, as a stepwise descending method with the variables that were significant factors in the univariate analyses, demonstrated that 30-day mortality was associated with malignancy (*p =* 0.003), higher CRP (*p =* 0.007), and higher UA/Cr (*p <* 0.001) (Table 4).

In univariate analyses, adverse composite outcome correlated with malignancy(*p =* 0.002), thrombophilia (*p =* 0.021), RV dysfunction (*p =* 0.002), higher respiratory rate (*p =* 0.002), higher PCO_2_ (*p <* 0.001),higher lactic acid (*p =* 0.001), elevated WBCs (*p =* 0.010), hyponatremia (*p <* 0.001), higher CRP (*p <* 0.001), higher PESI (*p =* 0.001) and ESC score (*p <* 0.001), high sPESI risk (*p =* 0.016) and higher UA/Cr(*p <* 0.001) However, age, sex, platelets, low blood pressure, cardiopulmonary disease and heart ratedid not affect composite outcome.

Multivariate logistic regression analysis, as a stepwise descending method, with the variables that were significant factors in the univariate analyses highlighted that composite outcome was associated with malignancy (*p =* 0.014), higher CRP (*p =* 0.013), and higher UA/Cr (*p =* 0.001) (Table 5).

### 3.4. Comparison with Existing Risk-Factor Prediction Models

**Figure 4:**
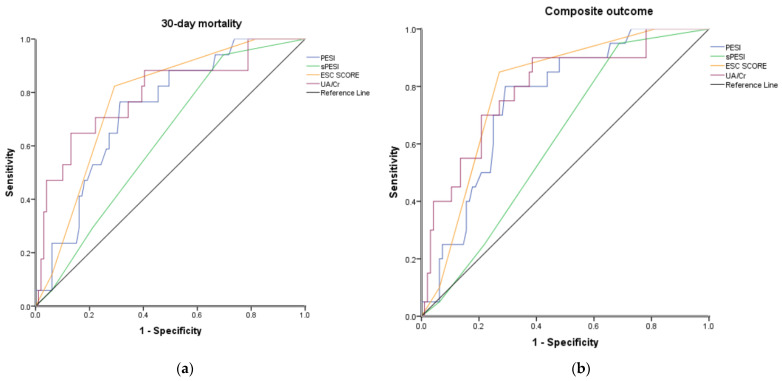
(**a**) ROC curves evaluating the diagnostic performance ofPESI, sPESI, ESC 2019 risk classification and uric acid to creatinine ratio as a predictor of 30-day mortality. (**b**) ROC curves evaluating the diagnostic performance ofPESI, sPESI, ESC 2019 risk classification and uric acid to creatinine ratio as a predictor of composite outcome.

A level of UA/Cr ≥7.636 showed 65% sensitivity, 87% specificity, 46% positive predictive value, 97% negative predictive value and 81% accuracy for 30-day mortality. In receiver operating characteristic analysis the area under the curve was 0.793 for 30-day mortality (95% CI 0.667–0.918, *p <* 0.001) and 0.793 for the composite outcome (95% CI 0.684–0.902, *p <* 0.001) (Figure 4). The performance of UA/Cr was similar with ESC Risk Classification for both 30-day mortality (AUC (95% CI) 0.793 (0.667–0.918) vs. 0.774 (0.670–0.877)) and composite outcome (0.793 (0.684–0.902) vs. 0.791 (0.697–0.884)), better than PESI score (0.734 (0.621–0.847) and (0.753 (0.651–0.855)), respectively; while simplified PESI was not diagnostic (0.622 (0.496–0.748) and (0.611 (0.495–0.727)), respectively (Table 6).

## 4. Discussion

In this study, we found that high serum UA/Cr level in subjects with pulmonary thromboembolism is an independent predictor of mortality and composite outcome. Higher serum UA/Cr level was significantly associated with PESI, simplified PESI and the ESC 2019 risk stratification. Moreover, serum UA/Cr levels were positively correlated with disease severity, duration of hospitalization and clinical and laboratory parameters. Therefore, it may be postulated that serum UA/Cr levels may be considered as a prognostic marker for patients with pulmonary thromboembolism.

One of the first studies investigating the relationship between serum UA levels and the risk of thromboembolic events in subjects with non-valvular AF was conducted by Numa et al. [23]. In this study the risk of a thromboembolic event was increased by 1.45 times independent of serum creatinine level in subjects with a high serum UA level. Similar to our results, Babaoglu et al. [24], found the lowest and the highest UA levels in subjects with low- and high-risk pulmonary thromboembolism, respectively. Serum UA levels were statistically significant in subjects with intermediate- or high-risk pulmonary thromboembolism, compared with subjects with low-risk pulmonary thromboembolism. However, contrary to our study the relationship between serum UA level and mortality was not evident in this study.

Elevated UA levels have long been known to be a poor prognostic sign in acute illness [25,26]. Previous studies have demonstrated that elevated UA levels in pulmonary hypertension correlate with the severity of disease and were independently related to mortality on multivariate Cox proportional hazards analysis [10,27]. Kaplan–Meier survival curves demonstrated that subjects with high serum UA had a significantly higher mortality rate than those with low serum UA. Moreover, the mean UA levels were higher in subjects with chronic thromboembolic pulmonary hypertension [11].

In the present study higher UA/Cr levels were significantly associated with PESI, simplified PESI, and ESC 2019 risk stratification. There could be several reasons for this finding. Patients with pulmonary embolism and lower UA levels had less cardiopulmonary comorbidities compared to patients with higher UA levels, as is shown in Table 1, which are included in PESI and simplified PESI scoring system, and ESC 2019 risk stratification. Increased respiratory rate in these patients may also have contributed to this correlation. Additionally, 2019 ESC risk classification includes right ventricular function and cardiac biomarkers, which may better reflect hemodynamic significance that is related with increased serum uric acid levels.

In multivariable analysis UA/Cr was an independent predictor of mortality (30-day and all cause) and composite outcome. Previous studies have also reported that elevated uric acid was predictive of PE severity and mortality [28,29]. This finding may be explained by the fact that in our cohort, patients with pulmonary embolism and elevated serum UA/Cr levels had increased PCO_2_, serum lactate, and d-dimers levels which may have had a significant impact on determining thrombolysis, severity of pulmonary embolism and oxygenation of the patients. UA, a byproduct of purine catabolism, is increased under the hypoxic conditions associated with diseases, such as obstructive sleep apnea, chronic obstructive pulmonary disease, and congestive heart failure [13,30,31]. In addition, UA levels have been shown to correlate negatively with cardiac output in patients with PE [28]. These findings suggest that hypoxia and decreased cardiac output in PE may elevate uric acid level, which correlates with the severity of PE and therefore with mortality and composite outcome.

Elevated serum UA level is associated with a wide range of diseases such as hypertension, chronic kidney disease, heart failure, and coronary artery disease, all these making it an important risk diagnostic tool [32]. Uric acid has been shown to be a useful biochemical marker of endothelial function, atherosclerosis, development of cardiovascular risk factors, and occurrence of cardiovascular events. It is worth mentioning that in patients with acute myocardial infarction, hyperuricemia is nowadays an established risk factor for adverse cardiovascular events, including cardiovascular mortality, independent of the presence of metabolic syndrome [33]. Recently there were established cut-off values for the optimal prediction of fatal myocardial infarction and all-cause mortality and increased serum UA values were able to independently predict the risk of premature acute myocardial infarction [34]. Finally, hyperuricemia is independently associated with an increased risk of both incident and recurrent atrial fibrillation. New evidence in this area was recently published [35] suggesting that not only serum UA levels above normal, but also a history of hyperuricemia contributes significantly to the risk of left atrial thrombosis.

To the best of our knowledge, there is no previous study evaluating the diagnostic role of UA/Cr as a biomarker in the evaluation of pulmonary embolism outcome. One of the limitations of our study was that it was a single-center, tertiary-care hospital study with a certain number of participants. The results of the present study should be validated by larger prospective cohort studies, in order to confirm the utilization of UA/Cr as a diagnostic tool during PE. A second limitation is that we assessed and followed-up the patients for a 30-day period after PE. Extended follow-up for a year could alter the results of our study. Thirdly, the evaluation of serum UA/Cr ratio was performed at a single moment in time.

## 5. Conclusions

In the present study of patients admitted with PE we demonstrated that serum UA/Cr ratio levels are associated with 30-day mortality, 30-day composite outcome, length of hospitalization, risk stratification, clinical and laboratory parameters and may be a useful biomarker for the identification of high-risk in patients at the time of the diagnosis of PE. Further prospective studies are needed for the evaluation of the diagnostic and prognostic role of this marker in larger populations with PE.

## Figures and Tables

**Table 1:** Baseline characteristics of patients with pulmonary embolism.

Variables	Total (*n* = 116)	UA/Create < 7.636 (*n* = 61)	UA/Create ≥ 7.636 (*n* = 55)	*p*
**Demographic Factors**				
Age, mean ± SD, years	53.47 ± 7.87	54.48 ± 7.32	52.35 ± 8.37	0.132
Male sex, *n* (%)	45 (38.8)	23 (37.7)	22 (39.9)	1.000
**Clinical Parameters**				
SBP, median (IQR)	135 (120–140)	137 (128–146)	135 (120–140)	0.091
Heart Rate, median (IQR)	100 (95–105)	100 (97–106)	101 (92–106)	0.956
Respiratory Rate, median (IQR)	28 (21–31)	21(18.5–23.5)	31(30–32)	**<0.001**
**Comorbidities**, *n* (%)				
Malignancy	27 (23.2)	11 (18.1)	16 (29.1)	0.190
Thrombophilia	21 (18.1)	12 (19.7)	9 (16.3)	0.470
DVT symptoms	29 (25)	17 (27.8)	12 (21.8)	0.286
Cardiopulmonary disease	33 (28.4)	8 (13.1)	22 (39.9)	**0.004**
**Laboratory Biomarkers**, median (IQR)				
Ua/Cr	7.59 (6.34–9.35)	6.40 (5.61–7.12)	9.5 (8.12–10.61)	**<0.001**
UA, mg/dL	6.8 (6.4–7.87)	6.5 (6.1–6.9)	7.7 (6.9–8.5)	**<0.001**
D-dimers, ng/mL	475 (350–720)	380 (300–640)	520 (390–740)	**0.013**
Oxygen saturation (%)	91 (88–93)	91 (88–93)	90 (87–93)	0.987
PaO_2_, mm Hg	73 (70–75)	73 (70–75.5)	73 (71.7–74.7)	0.640
PaCO_2_, mm Hg	36 (33.4–38.6)	34.3 (32.1–36.4)	37.9 (34.8–38.2)	**<0.001**
Lactic acid, mmol/L	1.73 (1.72–1.77)	1.73 (1.72–1.74)	1.74 (1.73–1.78)	**0.002**
WBC χ10^3^/μL	9.9 (8.94–12.5)	9.9 (8.8–12.06)	10.1 (9–12.97)	0.437
Platelets χ10^3^/μL	365 (295–457)	370 (300–467)	359 (280–447)	0.617
CRP, mg/dL	68 (12–100)	54 (11–87)	78 (14–138)	0.180
Na mEq/L	138 (135–140)	138 (136–139)	137 (134–139)	0.122
HSTPN, pg/mL	20 (14–40)	31 (14–45)	17 (14–29)	**<0.001**
**Pulmonary Embolism Classification Tools**				
ESC 2019 algorithm				
Low, *n* (%)	18 (15.5)	17 (27.9)	1 (1.8)	**<0.001**
Intermediate-low, *n* (%)	55 (47.4)	39 (63.9)	16 (29.1)	**<0.001**
Intermediate-high, *n* (%)	35 (30.2)	5 (8.2)	30 (54.5)	**<0.001**
High, *n* (%)	8 (6.9)	0 (0)	8 (13.8)	**0.002**
PESI, median (IQR)	113 (70–144)	76 (62–88)	144 (126–161)	**<0.001**
sPESI, median (IQR)	1 (0–1)	1 (0–1)	1 (1–2)	**0.019**
sPESI risk, high, *n* (%)	85 (73.2)	37 (60.6)	48 (87.2)	**0.001**

Bold indicates statistical significance. SBP: systolic blood pressure; DVT: deep vein thrombosis; Cardiopulmonary disease: chronic heart failure or pulmonary disease; UA/Cr: serum uric acid to creatinine ratio; UA: uric acid; PaO_2_: arterial partial pressure of oxygen; PaCO_2_: arterial partial pressure of carbon dioxide; WBC: white blood cells; CRP: c-reactive protein; HSTPN: high-sensitive troponin; ESC: European Society of Cardiology; PESI: pulmonary embolism severity index; sPESI: simplified PESI.

**Table 2:** Comparison of hospitalization outcomesin patients with low and high serum uric acid levels.

	Total	UA/Create < 7.636	UA/Create ≥ 7.636	*p*
Subjects, *n*	116	61	55	
Hospitalization days, median (IQR)	6 (5–8)	6 (5.5–8.5)	7 (5–11)	**0.003**
Mortality, *n* (%)	17 (14.6)	2 (3.2)	15 (27.3)	**<0.001**
Thrombolysis, *n* (%)	14 (12)	1 (1.6)	13 (23.6)	**<0.001**
Composite outcome, *n* (%)	20 (17.2)	2 (3.4)	18 (32.7)	**<0.001**

Bold indicates statistical significance.

**Table 3:** Predictors of hospitalization days.

	Univariate			Multivariate		
Variables	HR (95% CI)	Beta	*p*	HR (95% CI)	Beta	*p*
Age	0.002 (−0.085–0.090)	0.004	0.962			
Μale	1.135 (−0.258–2.528)	0.15	0.109			
Malignancy	2.199 (0.627–3.772)	0.251	**0.007**	1.373 (−0.416–3.162)	0.157	0.131
RV dysfunction	1.875 (0.542–3.209)	0.252	**0.006**	0.723 (−1.100–2.546)	0.097	0.433
Cardiopulmonary disease	0.260 (−1.261–1.780)	0.032	0.736			
Heart rate	−0.030 (−0.114–0.055)	−0.065	0.490			
Respiratory rate	0.108 (−0.010–0.226)	0.168	0.071	−0.154 (−0.343–0.036)	−0.239	0.11
SBP	−0.029 (−0.069–0.010)	−0.136	0.144			
Oxygen saturation	0.044 (−0.105–0.194)	0.055	0.558			
PaCO_2_	0.137 (−0.091–0.365)	0.111	0.237			
WBC	0.001 (0.000–0.001)	0.412	**<0.001**	0.001 (0.000–0.001)	0.35	**<0.001**
Na	−0.257 (−0.503–−0.012)	−0.191	**0.04**	0.103 (−0.163–0.369)	0.076	0.444
Hs TPNI	−0.012 (−0.052–0.027)	−0.057	0.545			
CRP	0.008 (0.000–0.015)	0.192	**0.039**	0.003 (−0.005–0.010)	0.064	0.507
Platelets	−0.006 (−0.011–−0.001)	−0.207	**0.026**	−0.001 (0.000–0.000)	−0.258	**0.002**
UA/Creat	0.574 (0.316–0.831)	0.382	**<0.001**	0.598 (0.209–0.987)	0.398	**0.003**
PESI score	0.024 (0.008–0.039)	0.275	**0.003**	0.006 (−0.021–0.034)	0.006	0.652
sPESI score	0.971 (−0.570–2.512)	0.116	0.214			
ESC Risk Classification	1.152 (0.329–1.975)	0.251	**0.006**	0.190 (−1.042–1.421)	0.041	0.761

Bold indicates statistical significance. RV: right ventricular; Cardiopulmonary disease: chronic heart failure or pulmonary disease; SBP: systolic blood pressure; PaCO_2_: arterial partial pressure of carbon dioxide; WBC: white blood cells; HSTPN: high-sensitive troponin; CRP: c-reactive protein; UA/Cr: serum uric acid to creatinine ratio; PESI: pulmonary embolism severity index; sPESI: simplified PESI; ESC: European Society of Cardiology.

**Table 4:** Predictors of 30-Day mortality.

	Univariate			Multivariate		
Variables	HR (95% CI)	Beta	*p*	HR (95% CI)	Beta	*p*
Age	0.974 (0.910–1.042)	0.026	0.444			
Male	0.890 (0.312–2.536)	−0.117	0.827			
Malignancy	0.145 (0.048–0.435)	−1.930	**0.001**	0.110 (0.026–0.470)	−2.206	**0.003**
RV dysfunction	0.204 (0.057–0.777)	−1.561	**0.019**	1.006 (0.034–29.614)	0.006	0.997
Cardiopulmonary disease	1.346 (0.405–4.476)	0.297	0.627			
Heart rate	1.008(0.946–1.074)	0.008	0.806			
Respiratory rate	1.166 (1.038–1.311)	0.154	**0.010**	0.873 (0.679–1.123)	−0.136	0.291
SBP	0.981 (0.952–1.010)	−0.019	0.200			
Oxygen saturation	0.990 (0.888–1.103)	−0.011	0.849			
PaCO_2_	1.467 (1.158–1.858)	0.383	**0.001**	1.172 (0.859–1.597)	0.158	0.316
WBC	1.000 (1.000–1.001)	0.0003	**0.004**	1.000 (1.000–1.001)	0.001	0.222
Na	0.658 (0.528–0.821)	−0.418	**<0.001**	1.122 (0.766–1.642)	0.115	0.555
HSTPNI	1.002 (0.973–1.032)	0.002	0.894			
CRP	1.008 (1.003–1.013)	0.008	**0.001**	1.009 (1.002–1.015)	0.009	**0.007**
Platelets	1.000 (1.000–1.001)	0.001	0.120			
UA/Cr	1.575 (1.264–1.963)	0.454	**<0.001**	1.620 (1.245–2.108)	0.482	**<0.001**
PESI score	1.020 (1.006–1.033)	0.020	**0.003**	0.999 (0.965–1.034)	−0.001	0.951
sPESI score	1.577 (0.865–2.874)	0.455	0.137			
ESC Risk Classification	3.559 (1.696–7.469)	1.269	**0.001**	2.215 (0.510–9.618)	0.795	0.289

Bold indicates statistical significance. RV: right ventricular; Cardiopulmonary disease: chronic heart failure or pulmonary disease; SBP: systolic blood pressure; PaCO_2_: arterial partial pressure of carbon dioxide; WBC: white blood cells; HSTPN: high-sensitive troponin; CRP: c-reactive protein; UA/Cr: serum uric acid to creatinine ratio; PESI: pulmonary embolism severity index; sPESI: simplified PESI; ESC: European Society of Cardiology.

**Table 5:** Predictors of adverse composite outcome.

	Univariate			Multivariate		
Variables	HR (95% CI)	Beta	*p*	HR (95% CI)	Beta	*p*
Age	0.995 (0.935–1.058)	−0.005	0.868			
Male	0.733 (0.277–1.940)	−0.310	0.532			
Malignancy	0.215 (0.078–0.597)	−1.536	**0.003**	0.214 (0.062–0.735)	−1.540	**0.014**
RV dysfunction	0.162 (0.045–0.590)	−1.818	**0.006**	1.915 (0.162–22.627)	−0.650	0.606
Cardiopulmonary disease	0.913 (0.318–2.622)	−0.091	0.866			
Heart rate	0.976 (0.919–1.036)	−0.025	0.421			
Respiratory rate	1.179 (1.054–1.317)	0.164	**0.004**	0.926 (0.770–1.112)	−0.077	0.411
SBP	0.992 (0.965–1.020)	−0.008	0.558			
Oxygen saturation	1.013 (0.908–1.131)	0.013	0.811			
PaCO_2_	1.416 (1.148–1.746)	0.348	**0.001**	1.123 (0.877–1.439)	0.116	0.357
WBC	1.000 (1.000–1.001)	0.001	**0.012**	1.000 (0.998–1.002)	0.001	0.178
Na	0.703 (0.577–0.856)	−0.353	**<0.001**	1.067 (0.790–1.844)	0.065	0.673
HSTPNI	1.015 (0.988–1.042)	0.014	0.283			
CRP	1.007 (1.003–1.012)	0.007	**0.002**	1.007 (1.001–1.012)	0.007	**0.013**
Platelets	1.000 (1.000–1.000)	0.000	0.199			
UA/Cr	1.537 (1.248–1.894)	0.430	**<0.001**	1.521 (1.211–1.908)	0.419	**<0.001**
PESI score	1.022 (1.009–1.035)	0.021	**0.001**	1.001 (0.973–1.030)	0.001	0.951
sPESI score	1.491 (0.846–2.627)	0.399	0.167			
ESC Risk Classification	3.926 (1.899–8.117)	1.368	**<0.001**	2.100 (0.822–5.365)	0.742	0.121

Bold indicates statistical significance. RV: right ventricular; Cardiopulmonary disease: chronic heart failure or pulmonary disease; SBP: systolic blood pressure; PaCO_2_: arterial partial pressure of carbon dioxide; WBC: white blood cells; HSTPN: high-sensitive troponin; CRP: c-reactive protein; UA/Cr: serum uric acid to creatinine ratio; PESI: pulmonary embolism severity index; sPESI: simplified PESI; ESC: European Society of Cardiology.

**Table 6:** The performance of each risk factor prediction model with respect to predicting 30-day mortality and composite outcome.

	30-Day Mortality	Composite Outcome
Predictors	AUC (95% CI)	AUC (95% CI)
PESI score	0.734 (0.621–0.847)	0.753 (0.651–0.855)
PESI score, class I–V	0.738 (0.629–0.847)	0.763 (0.664–0.861)
sPESI score	0.622 (0.496–0.748)	0.611 (0.495–0.727)
sPESI score, class 0–1	0.622 (0.495–0.749)	0.631(0.513–0.749)
ESC 2019 Risk Classification	0.774 (0.670–0.877)	0.791 (0.697–0.884)
UA/Cr	0.793 (0.667–0.918)	0.793 (0.684–0.902)

PESI: pulmonary embolism severity index; sPESI: simplified PESI; ESC: European Society of Cardiology; UA/Cr: serum uric acid to creatinine ratio.

## Data Availability

The datasets generated during and/or analyzed during the current study are available from the corresponding author on reasonable request.
